# Physicochemical, Rheological, and Sensory Characterization of Gluten-Reduced Cookies Enriched with Amaranth and Chickpea Flours

**DOI:** 10.3390/foods15152751

**Published:** 2026-08-05

**Authors:** Stalin Aldair De la Cruz Sarchi, Leslie Josseline Pozo Alvear, Carlos Alberto Rivas Rosero, Freddy Giovanny Torres Mayanquer, Jorge Ivan Mina Ortega, Guillermo Alexander Jácome Sarchi

**Affiliations:** 1 Carrera de Alimentos, Universidad Politécnica Estatal del Carchi, Tulcán 040102, Ecuador; stalin.delacruz@upec.edu.ec (S.A.D.l.C.S.); leslie.pozo@upec.edu.ec (L.J.P.A.); carlos.rivas@upec.edu.ec (C.A.R.R.); freddy.torres@upec.edu.ec (F.G.T.M.); jorge.mina@upec.edu.ec (J.I.M.O.); 2 Grupo de Investigación Agricultura Sostenible (GIAS), Carrera de Agropecuaria, Universidad Politécnica Estatal del Carchi, Tulcán 040102, Ecuador

**Keywords:** *Amaranthus caudatus*, *Cicer arietinum*, Mixolab, dough rheology, texture profile analysis, principal component analysis, viscoelastic network

## Abstract

The global transition toward plant-based diets necessitates the development of sustainable, nutritionally enriched, and gluten-reduced bakery products. This study evaluated the techno-functional, nutritional, and sensory impacts of substituting wheat flour at levels of 30%, 50%, and 70% with a binary blend of amaranth (*Amaranthus caudatus*) and chickpea (*Cicer arietinum*) flours in short-dough cookies. Dough’s thermo-mechanical behavior was characterized using the Mixolab Chopin+ protocol and correlated with Texture Profile Analysis (TPA) and consumer acceptability (*n* = 60) via Principal Component Analysis (PCA). Results revealed that the 70% substitution level (T3) significantly modified dough rheological behavior; the thermodynamic dilution of the gluten network and competitive hydration led to reduced dough stability (5.40 min) and restricted starch gelatinization (C3 = 0.916 Nm). Despite these rheological limitations, T3 demonstrated the highest protein density among the formulations tested, achieving 14.61% crude protein and 2.18% ash. Notably, the significant increase in instrumental hardness (68.09 N) in T3 corresponded with the highest overall acceptability score (4.07 out of 5.0). Multivariate analysis indicated that dough rheological instability does not necessarily limit final product acceptability in non-fermented matrices. These findings provide useful insights for the rational design of high-protein, plant-based functional snacks.

## 1. Introduction

The global transition toward plant-based diets and sustainable nutrition has driven the urgent need for nutritionally enriched and gluten-reduced bakery products [1]. Cookies, due to their long shelf life and widespread consumption, have become an ideal matrix for nutritional enhancement through the incorporation of alternative, non-conventional flours [2]. In recent years, the development of vegan and protein-enriched cookies has gained significant traction, requiring a delicate balance between structural integrity and sensory acceptability [3]. The use of legume-derived flours and plant protein concentrates has proven to be an effective strategy to elevate the techno-functional profile of these short-dough systems while addressing the growing consumer demand for clean-label and high-protein snacks [4].

Beyond nutritional improvements, the incorporation of these non-conventional flours fundamentally disrupts the microstructural dynamics of the dough [5]. The native starch granules of pseudocereals, such as amaranth, exhibit distinctive pasting behaviors and morphological traits that alter water distribution when competing with legume proteins [6]. Concurrently, the introduction of globular proteins and insoluble dietary fibers from chickpea significantly modifies the viscoelastic matrix, restricting starch swelling and gelatinization during thermal transitions [7]. Recent studies emphasize that understanding the complex interactions within this starch–protein matrix is critical, as these microstructural and rheological shifts directly dictate the mechanical properties and final texture of the baked product [8].

To accurately evaluate these complex structural transitions, traditional empirical rheology tools—such as the Farinograph, which operates strictly at a constant low temperature—often fall short in predicting the final quality of non-conventional matrices [9]. In contrast, the Mixolab system provides useful thermo-mechanical profiling by applying controlled heating and cooling phases combined with mechanical mixing in a single test [10]. This integration allows for a better understanding of dough behavior in composite matrices, capturing the effects of protein weakening and starch pasting that traditional cold-mixing methods typically overlook [11]. Cookies represent an ideal matrix for nutritional reformulation due to their low-moisture, non-fermented structure, which accommodates nutrient-dense alternative flours such as legumes and pseudocereals while maintaining acceptable product quality [12,13,14,15]. Utilizing regional raw materials like chickpea and amaranth offers a promising strategy for developing enriched bakery products with high local added value [16,17]. However, high-level substitution fundamentally alters the internal dynamics of the dough. The techno-functional response is governed by the complex redistribution of water among starch, gluten, and non-gluten proteins [18,19,20]. In these systems, the viscoelastic weakening of gluten combined with water competition from globular storage proteins (from chickpea) and distinctive starches (from amaranth) significantly modifies protein–protein interactions and starch gelatinization [21,22,23,24,25]. Although these thermo-mechanical transitions are critical determinants of final cookie texture, the specific physical interactions within massive ternary substitution blends remain insufficiently characterized.

Recent research on composite cookie formulations has shown that partial substitution of wheat flour can enhance nutritional value while altering physicochemical and sensory properties; however, these studies primarily evaluate end-product attributes without fully addressing the underlying rheological mechanisms governing dough behavior [26]. Similarly, evidence from composite bread systems has provided valuable insight into dough rheology and starch–protein interactions [27,28]. Nevertheless, these findings serve primarily as mechanistic support rather than direct predictors of cookie performance, given the fundamental differences in processing conditions and structure development between fermented bread systems and short-dough products [29].

To overcome these descriptive limitations, thermo-mechanical analysis using the Mixolab system enables simultaneous monitoring of protein weakening and starch gelatinization under controlled processing conditions [23]. However, limited evidence is available regarding the integration of Mixolab parameters, Texture Profile Analysis (TPA) descriptors, and multivariate statistical approaches such as Principal Component Analysis (PCA) in wheat–amaranth–chickpea ternary cookie systems to elucidate the relationship between dough behavior and final product texture.

Therefore, the objective of this study was to evaluate the effect of a wheat–amaranth–chickpea ternary matrix on dough thermo-mechanical behavior and to determine the association between Mixolab parameters, particularly C3 and C5 torques, and instrumental texture attributes through multivariate analysis. This approach contributes to the development of exploratory descriptive frameworks for nutritionally enhanced reduced-gluten cookie formulations.

## 2. Materials and Methods

### 2.1. Raw Materials and Flour Preparation

A single commercial lot (harvest year 2025) of amaranth grains (*Amaranthus caudatus* L., cv. Arcoiris) and chickpea grains (*Cicer arietinum* L., cv. Kabuli) was acquired from local agricultural suppliers in Tulcán, Carchi, Ecuador. The raw materials were processed within one week of acquisition. Refined wheat flour (WF), complying with the NTE INEN 616 standard [30], and other baking ingredients—including eggs, sugar, baking powder, vanilla extract, and unsalted butter—were purchased from a local supermarket.

To standardize processing conditions, the grains were spread in a uniform layer thickness of approximately 1.5 cm and dehydrated in a convection oven at a constant temperature of 80 °C for a brief period of 20 min before milling. Given the initial moisture content of the commercial dry grains (~12%), this short thermal treatment successfully reduced the superficial moisture to a final range of 8.5–9.0% (as confirmed in Section 3.1). This step was applied specifically to ensure efficient milling, prevent microbial development, and partially inactivate lipolytic enzymes and anti-nutritional factors without causing complete starch gelatinization or significant protein denaturation.

The dried grains were milled using a laboratory hammer mill (IKA-Werke GmbH & Co., Staufen, Germany) operated at a rotor speed of approximately 3000 rpm with a continuous manual feed rate and equipped with a 500 µm standardized sieve to ensure uniform particle size. The resulting amaranth flour (AF) and chickpea flour (CF) were packed in hermetically sealed polyethylene bags and stored at 15 ± 2 °C with a relative humidity of 50 ± 5% to preserve flour quality and minimize oxidative deterioration prior to dough formulation for a maximum of 15 days.

### 2.2. Physicochemical Characterization

Nutritional composition of raw flours was determined following AOAC standard methods [31]: moisture (Method 925.10), total ash (Method 923.03), crude fat (Method 945.16), and total crude protein (Method 981.10). A nitrogen-to-protein conversion factor of 6.25 was applied for comparative purposes among formulations. Titratable acidity was determined by homogenizing a 10 g flour sample in 100 mL of distilled water (1:10 ratio), followed by titration with a standardized 0.1 N NaOH solution using phenolphthalein as an indicator until a faint persistent pink endpoint (pH~8.1) was achieved. The results were calculated and expressed as a percentage of lactic acid equivalents. The wheat flour base was verified against NTE INEN 616 [30].

### 2.3. Experimental Design and Formulations

A completely randomized design (CRD) was implemented to evaluate the partial substitution of wheat flour (WF) with a 1:1 blend of AF and CF. Three independent batches were produced for each formulation, where each batch represented an independent experimental unit. Based on preliminary formulation screening, three substitution levels were selected to represent increasing technological constraints associated with wheat flour substitution and starch–protein matrix restructuring:

- T1: 70% WF + 15% AF + 15% CF;
- T2: 50% WF + 25% AF + 25% CF;
- T3: 30% WF + 35% AF + 35% CF.

Fixed ingredients (expressed as % of total dough weight) were butter (21.51%), sucrose (17.21%), whole eggs (8.26%), vanilla essence (0.86%), and baking powder (0.52%).

### 2.4. Dough Thermo-Mechanical Properties (Mixolab Analysis)

The thermo-mechanical behavior was evaluated using a Mixolab (Chopin Technologies, *Villeneuve la Garenne,* France) following the Chopin+ protocol (AACC Method 54-60.01) [32]. Although Mixolab is traditionally optimized for bread dough, it serves as a critical diagnostic tool to assess the impact of wheat flour substitution on the thermo-mechanical properties of composite matrices, capturing protein weakening and starch gelatinization transitions that fundamentally dictate the final structure and hardness of the short-dough cookies. A dough sample prepared from 75 g of flour on an equivalent basis was analyzed, and water addition was adjusted according to flour moisture content to achieve a constant 14% moisture basis. Each formulation was analyzed in triplicate using independent dough preparations.

The analysis was performed under controlled mechanical (80 rpm) and thermal conditions according to the Chopin+ protocol, generating torque profiles related to dough development, protein weakening, starch gelatinization, enzymatic activity, and retrogradation behavior. The primary torque parameters (C1 to C5) were recorded, and derived parameters were calculated to describe mechanical dynamics: C2-C1 (protein weakening index), C3-C2 (gelatinization development index), and C5-C4 (retrogradation tendency).

### 2.5. Cookie Manufacturing

Cookies were prepared following a standardized short-dough creaming method to ensure batch-to-batch reproducibility. The creaming phase was performed using a KitchenAid Artisan planetary mixer (KitchenAid, St. Joseph, MI, USA), equipped with a flat beater attachment. Butter and sucrose were creamed at medium speed for 3 min until a homogeneous paste was formed. Whole eggs and vanilla essence were then added and mixed for an additional 2 min. Subsequently, the flour blends (T1, T2, or T3) and baking powder were gradually incorporated. The dough was mechanically kneaded for exactly 10 min to ensure uniform hydration and then allowed to rest for 30 min at room temperature.

The rested dough was sheeted to a uniform thickness of 5 mm using a standardized rolling pin with guide rings and cut into circular discs (50 mm diameter). Cookies were placed on a perforated aluminum tray lined with parchment paper. Baking was carried out in a temperature-controlled gas oven preheated to 180 °C. The trays were baked for 20 min. Post-baking, cookies were allowed to cool at room temperature (20 °C) on a wire rack for 30 min before being packed in high-density polyethylene (HDPE) Ziplock bags and stored in a desiccator prior to instrumental and sensory analyses.

### 2.6. Quality Attributes

#### 2.6.1. Physicochemical Properties

Proximate composition was analyzed according to AOAC standard methods [31]. The pH of the baked cookies was determined using a calibrated digital pH meter (SevenDirect SD23, Mettler Toledo, Columbus, OH, USA) following a standardized solid–liquid extraction protocol based on NTE INEN 2085 [33]. Briefly, 10 g of pulverized cookie sample was homogenized in 100 mL of distilled water (yielding a 1:10 cookie-to-water ratio) using continuous magnetic stirring for 5 min. The suspension was then allowed to equilibrate for 30 min at room temperature (20 ± 1 °C) to ensure adequate solubilization of the components before recording the measurement. All analytical determinations were performed in triplicate for each independent batch.

#### 2.6.2. Instrumental Texture (TPA)

Instrumental texture analysis was performed 24 h after baking to allow moisture equilibration. Measurements were conducted under controlled environmental conditions (25 °C and 50% relative humidity) using a Brookfield CT3 Texture Analyzer (AMETEK Brookfield, Middleboro, MA, USA) equipped with a TA41 probe (6 mm diameter).

A two-cycle compression test was applied according to the Texture Profile Analysis (TPA) procedure. Ten cookies from each formulation (approximately 3–4 cookies per independent batch) were analyzed individually, and mean values were used for statistical comparison. The instrumental parameters were trigger load 0.07 N, test speed 8 mm/s, and target deformation 8 mm.

The Texture Profile Analysis (TPA) was selected due to its ability to provide a comprehensive structural characterization of the ternary cookie matrix. In this context, the initial peak force observed during the first compression cycle was recorded as the indicator of cookie fracturability (or snap force), consistent with standardized procedures for assessing the structural integrity of short-dough products [19,20].

### 2.7. Sensory Evaluation

The evaluation was conducted with 60 untrained consumers (predominantly young adult university students and staff) selected according to recommendations for affective consumer acceptance tests [34]. Participants were recruited based on their regular consumption of bakery products and the explicit absence of food allergies. Because the sensory evaluation was strictly limited to standard hedonic testing of cookies made with safe, commercially available food-grade ingredients, formal Institutional Ethics Committee approval was exempted. However, informed consent was obtained from all subjects prior to participation.

The tasting sessions were conducted in well-lit, odor-free university rooms. The three cookie formulations (T1, T2, and T3) were presented simultaneously on white plates, coded with randomized three-digit alphanumeric numbers to prevent bias. Panelists evaluated attributes including color, odor, flavor, texture, and overall acceptability using a 5-point hedonic scale (1 = “dislike extremely”; 3 = “neither like nor dislike”; 5 = “like extremely”). Purified drinking water at room temperature was provided to the panelists to cleanse their palates between tasting each sample.

### 2.8. Statistical Analysis

All experiments were performed using three independent batches per formulation. Data distribution was verified using the Shapiro–Wilk test, and homogeneity of variance was assessed using Levene’s test.

Differences among formulations were evaluated by one-way analysis of variance (ANOVA), followed by Tukey’s honestly significant difference (HSD) test (*p* < 0.05). Non-parametric sensory data (repeated measures) were analyzed using the Friedman test, followed by a Wilcoxon signed-rank post hoc test with Bonferroni correction.

Pearson correlation coefficients (r) were calculated using batch mean values to explore linear associations among physicochemical, rheological, textural, and sensory attributes. Due to the limited number of formulation points, this approach was strictly utilized as an exploratory and descriptive visualization rather than a predictive tool.

Principal Component Analysis (PCA) was similarly performed as an exploratory multivariate approach to visualize associations among thermo-mechanical, compositional, and texture variables using R software (version 4.3.0) with the FactoMineR and ggplot2 packages. Batch means were considered the experimental units for statistical comparisons to avoid pseudoreplication. Statistical significance was established at an alpha level of 0.05.

## 3. Results

### 3.1. Proximate Composition of Raw Materials

The nutritional and physicochemical profiles of the non-conventional flours used to develop the ternary blends are presented in Table 1. The characterization confirmed that both amaranth flour (AF) and chickpea flour (CF) serve as potent fortification agents due to their dense nutrient composition. Amaranth flour exhibited a superior protein content of 18.18 ± 0.12%, which was significantly higher (*p* < 0.05) than the 16.79 ± 0.15% found in chickpea flour, both of which vastly outperformed the baseline protein content of the refined wheat flour control (10.50).

Furthermore, AF showed higher concentrations of crude fat (6.23 +/− 0.15%) and total ash (4.85 +/− 0.08%) compared to CF, highlighting its role as a significant source of essential lipids and minerals for the final cookie matrix. Regarding stability markers, the moisture levels for both flours remained well below the 10% safety threshold, typically required to maintain flour quality during storage.

Titratable acidity, used as a basic indicator of general flour freshness, was significantly lower in chickpea flour (0.02 ± 0.01%) than in amaranth flour (0.06 ± 0.01%). These values confirm that the dehydration process effectively stabilized the flours, maintaining a low acidity profile prior to the manufacturing process.

### 3.2. Dough Thermo-Mechanical Profiling (Mixolab)

**Figure 1:**
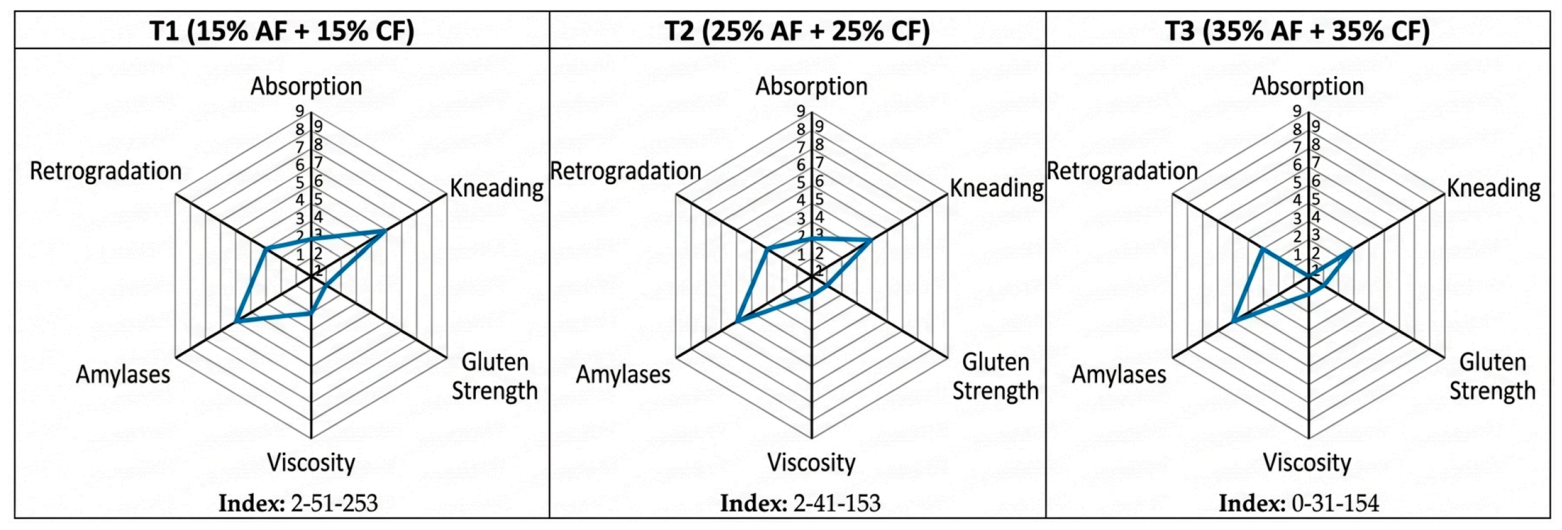
Mixolab Profiler radar charts and functional indices of ternary flour blends (T1, T2, and T3) at different substitution levels of amaranth and chickpea flours.

The thermo-mechanical properties of the ternary flour blends, determined using the Mixolab system (Chopin+ protocol), are summarized in Table 2. The substitution of wheat flour with increasing levels of amaranth (AF) and chickpea (CF) flours significantly (*p* < 0.05) modified the rheological behavior of the dough across all stages. These changes are visually synthesized in the Mixolab Profiler radar charts (Figure 1), which provide a functional “fingerprint” of the dough’s performance.

Water absorption showed a notable reduction as the substitution level increased, dropping from 56.3 ± 0.45% (T1) to 49.7 ± 0.52% (T3). This trend is quantitatively supported by the Absorption Index, which plummeted from 2 (T1) to 0 (T3). This indicates a severe disruption in the hydration kinetics, as the globular proteins and fibers from the alternative flours do not sequester water in the same manner as the wheat glutenin/gliadin complex.

During the initial mixing phase, C1 torque—which represents the maximum torque during mixing—showed a slight but significant decrease as substitution increased, from 1.144 ± 0.02 Nm (T1) to 1.108 ± 0.01 Nm (T3). Furthermore, dough stability exhibited a sharp decline. T1 (30% substitution) presented a stability of 9.80 ± 0.15 min, which decreased to 5.00 ± 0.20 min for T2 and 5.40 ± 0.12 min for T3. The Mixing Index, shifting from 5 (T1) to 3 (T3), supports the hypothesis of a “dilution effect” on the gluten network, rendering the dough more susceptible to mechanical shear.

The thermal processing stages also revealed significant changes. The C3 torque (starch gelatinization) reached its lowest value in T3 (0.916 ± 0.03 Nm) compared to T1 (1.291 ± 0.05 Nm). This suggests that the high concentration of alternative proteins and fibers competes for available water, restricting starch granule swelling. Finally, the decrease in C5 torque (retrogradation), reflected in the shifting Profiler indices from 2-51-253 (T1) to 0-31-154 (T3), suggests that the enriched dough matrices may exhibit slower retrogradation rates.

### 3.3. Physicochemical and Nutritional Characterization of Cookies

The nutritional profile of the formulated cookies, as detailed in Table 3, demonstrated a significant (*p* < 0.05) and progressive increase in all measured proximate components (protein, fat, and ash) as the substitution level of the ternary matrix rose. The crude protein content reached its highest level in the T3 treatment with 14.61 ± 0.18%, significantly surpassing T2 (13.67 ± 0.12%) and T1 (13.47 ± 0.15%). This enrichment confirms that the combination of amaranth and chickpea flours effectively fortifies the cookies. While a 100% wheat control was not experimentally evaluated in this study, this final protein value (14.61%) represents a substantial nutritional enhancement when compared to standard literature reference values for conventional wheat short-dough cookies, which typically range between 5% and 7% protein [4,12].

The mineral content (ash) and crude fat also showed significant upward trends, reflecting the high nutritional quality of the alternative raw materials used. Moisture content increased from 7.16 ± 0.13% (T1) to 8.18 ± 0.15% (T3); however, all values remained well within the maximum limit established by the national regulatory standard (NTE INEN 2085) for commercial cookie quality. Furthermore, the pH values showed a slight but significant increase with higher substitution levels, ranging from 6.72 ± 0.02 to 6.82 ± 0.03. These values are consistent with the requirements for bakery products under the Ecuadorian standard NTE INEN 2085 [33], indicating that the ternary blends maintain a stable chemical environment suitable for the development of desirable sensory characteristics during baking.

### 3.4. Instrumental Texture Profile Analysis (TPA)

Instrumental texture parameters, which are decisive for the structural integrity and quality perception of cookies, were significantly affected (*p* < 0.0001) by the substitution of wheat flour with the amaranth–chickpea matrix. The results of the double compression test (TPA), summarized in Table 4, reveal a linear and robust increase in all mechanical attributes as the inclusion of alternative flours rose from 30% to 70%.

Hardness C1 and fracturability, which showed identical numerical values in this matrix (because the brittle short-dough cookies fracture at the absolute peak force of the first compression cycle before reaching maximum target deformation), exhibited a dramatic increase from 32.31 ± 1.28 N (T1) to 68.09 ± 1.54 N (T3). This structural hardening is a direct mechanical consequence of the starch–protein matrix restructuring. The partial replacement of viscoelastic wheat proteins with globular proteins and insoluble fibers from amaranth and chickpea hinders the formation of an expansive crumb during baking. Instead, a more compact and rigid protein–starch matrix is formed, which requires significantly higher peak force during the initial fracture.

The chewiness of the cookies followed an even more pronounced trend, rising exponentially from 3.67 ± 1.20 mJ (T1) to 25.47 ± 4.48 mJ (T3). This increase in the energy required for mastication is associated with the higher moisture retention and density of the T3 treatment. These findings are consistent with the Mixolab stability data (Section 3.2), where the reduced stability of the T3 dough translated into a final product with greater resistance to mechanical deformation. From a technological standpoint, while the T3 cookies are significantly harder, this rigidity contributes to the structural integrity of a product with a 70% reduction in wheat flour.

### 3.5. Sensory Perception and Consumer Acceptance

The sensory acceptability of enriched bakery products is an essential indicator of their market potential. The sensory profile of the formulated cookies, as summarized in Table 5, revealed that specific attributes (color, odor, flavor, and texture) showed significant differences (*p* < 0.05) as the substitution level of the ternary matrix increased. Due to the ordinal nature of the sensory scores and the repeated-measures design (the same panelists evaluated all samples), a Friedman non-parametric test was applied.

The T3 treatment (70% substitution) achieved the highest median scores across the evaluated attributes: color, odor, flavor, and texture. Notably, the overall acceptability showed no significant differences among the three treatments (*p* = 0.072). This is a highly positive outcome, indicating that massive wheat substitution up to 70% did not negatively affect the overall consumer perception, which remained uniformly high (median = 4.0) across all formulations. The high median scores in color (4.0) and flavor (4.0) for T3 could be linked to enhanced Maillard reactions promoted by the higher protein content (14.61%, Section 3.3) and the specific amino acid profiles of amaranth and chickpea. These components facilitate the development of attractive golden-brown pigments and complex aromatic compounds during baking, which are highly valued by consumers.

Crucially, the sensory perception of texture provided essential context for the instrumental findings (Section 3.4). Although the TPA analysis identified T3 as the mechanically hardest formulation (68.09 N), the sensory panel awarded it the highest median acceptability score for the texture attribute (4.0). While our hedonic evaluation did not utilize descriptive profiling to specifically quantify attributes such as crispiness or crunchiness, this outcome suggests that the increased mechanical rigidity was not perceived as a sensory defect by the consumers. This indicates that a 70% substitution level maximizes the nutritional density of the cookies while maintaining a highly acceptable organoleptic profile.

### 3.6. Multivariate Analysis: Linking Dough Rheology to Final Product Quality

To explore the macroscopic interrelationships between dough thermo-mechanical properties, physicochemical composition, instrumental texture, and sensory perception, an exploratory Principal Component Analysis (PCA) and Pearson correlation matrix were applied to the experimental dataset (Table S1 and Figure S1, Supplementary Materials). While the limited number of formulation points restricts definitive mechanistic inferences, this exploratory approach indicates that the first two principal components (PC1 and PC2) accounted for the majority of the total variance, effectively separating the formulations along the primary axis based on their substitution levels.

**Figure 2:**
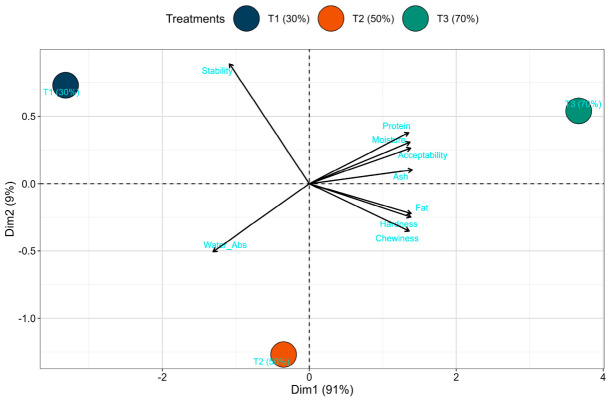
Principal Component Analysis (PCA) biplot illustrating the multivariate associations among dough thermo-mechanical parameters, physicochemical composition, instrumental texture attributes, and sensory acceptability of the formulated cookies at different substitution levels (T1: 30%, T2: 50%, and T3: 70%).

The PCA biplot (Figure 2) reveals a clear spatial distribution of the treatments. The low-substitution formulation (T1) is positioned in the quadrant strongly associated with Mixolab rheological parameters, specifically water absorption and dough stability. This confirms that T1 retains a more traditional wheat-like functional behavior. Conversely, the high-substitution treatment (T3, 70%) is diametrically opposed along PC1, showing a robust positive association with instrumental hardness, crude protein content, ash, and sensory acceptability scores.

Based on batch mean values, the Pearson correlation analysis further elucidates these macro-structural relationships. A significant negative linear correlation was observed between dough stability (Mixolab) and instrumental hardness (TPA), confirming that the starch–protein matrix restructuring by alternative proteins is associated with a more rigid baked matrix. Most importantly, instrumental hardness exhibited a strong positive correlation with sensory texture and overall acceptability. Within the limited formulations tested in this exploratory visualization, the mechanical rigidity imparted by the amaranth and chickpea flours was consistently aligned with higher consumer acceptability scores. Ultimately, this exploratory multivariate approach suggests that while progressive substitution modifies conventional dough rheology, it synergistically enhances the nutritional density and sensory profile of the final product.

## 4. Discussion

### 4.1. Dough Rheology and Microstructural Matrix Dynamics

The substitution of wheat flour with a binary blend of amaranth and chickpea flours profoundly altered the thermo-mechanical behavior of the dough, directly impacting the final product’s quality. As observed by the Mixolab profiling (Section 3.2), dough stability decreased drastically from 9.80 min in the formulation (T1) to 5.40 min in the highly substituted matrix (T3). This phenomenon aligns with recent findings in the literature [35,36], which report that the incorporation of gluten-free pseudocereals and legumes significantly decreases dough stability due to the thermodynamic dilution of the glutenin–gliadin network. We hypothesize that a reduction in wheat proteins restricts the formation of a continuous viscoelastic web, which could render the dough more susceptible to mechanical shear during mixing [37]. Recent optimization studies on composite flours have confirmed that adding amaranth and chickpea exceeding 30% disrupts the continuous protein phase, causing rapid mechanical weakening [38,39].

Furthermore, the significant drop in water absorption from 56.3% (T1) to 49.7% (T3) suggests that the substitution of wheat flour with amaranth and chickpea flours alters the dough’s hydration dynamics. This trend may be explained by the dilution of the gluten network and potential competitive water binding between the residual glutenin–gliadin complex and the non-gluten proteins and fibers from the alternative flours, which could restrict starch swelling during thermal processing. While this competitive hydration is a plausible explanation for the reduction in C3 torque, these observations represent a functional outcome of the composite matrix, and further studies focusing on specific protein–water interactions would be required to definitively decouple these rheological mechanisms. Somsap and Tepsorn [38] observed a similar competitive hydration in chickpea composite flours, where the native starch failed to fully gelatinize, which supports the hypothesis that a physical barrier may be created by legume globulins and fibers [40].

### 4.2. Nutritional Enhancement and Texture Perception

The core objective of formulating the ternary blends was the nutritional enrichment of the cookies, which was successfully validated by the significant increase in protein (14.61%) and ash (2.18%) in the T3 treatment. Legumes like chickpea are recognized in the literature for their high lysine content, which can complement the sulfur-containing amino acids in amaranth [41,42]. While specific amino acid scoring or digestibility assays (e.g., PDCAAS) were not performed in the present study, the literature indicates that this binary combination provides a theoretical improvement in overall protein quality compared to standard wheat flour alone.

However, a critical dynamic emerges when correlating the nutritional enrichment with the physical structure of the cookie. As previously discussed, the disruption of the gluten network and the reduced water availability led to a highly significant increase in instrumental hardness (68.09 N in T3). Typically, in conventional bakery science, extreme hardness is considered a quality defect. Yet, the sensory evaluation (Section 3.5) revealed that the instrumentally hardest cookie (T3) received the highest acceptability score for texture (median = 4.0) from the panelists.

This divergence is crucial for product development. It indicates that the mechanical ‘hardness’ registered by the TPA did not negatively impact the panelists’ perception; rather, it translated into a highly acceptable texture profile for this type of short-dough cookie [43]. While previous studies [42,44] have suggested that high-protein legume cookies exhibit altered fracture mechanics, within the scope of our evaluation, the results suggest that the structural rigidity observed at the 70% substitution level did not detract from, and was concurrent with, high organoleptic acceptance.

### 4.3. Multivariate Validation of Product Quality

The Principal Component Analysis (Figure 2) effectively and visually summarizes the success of the 70% substitution level, aligning perfectly with recent approaches for the multivariate integration of functional and compositional transitions in gluten-free matrices [45]. The spatial segregation of T3 from T1 confirms that the fortification strategy fundamentally alters the product identity. While T1 is anchored by conventional wheat properties (high stability, high hydration), T3 represents a new paradigm of functional cookies, strongly correlated with high protein density, high fat (contributing to sensory mouthfeel), and superior overall acceptability.

Crucially, the increased sensory scores for color and flavor in T3 are hypothesized to be associated with its higher protein content, which provides a greater concentration of amino acids necessary for the Maillard reaction [44,45]. While specific colorimetric indices or volatile profiles were not measured in this study, the literature widely reports that this non-enzymatic browning generates the attractive golden pigments and complex roasted flavor profiles that the panelists likely favored. Ultimately, the multivariate approach demonstrates that the inclusion of 70% amaranth and chickpea flours does not merely degrade conventional dough rheology; rather, it synergistically creates a novel, highly nutritious, and sensorially superior functional food, validating the viability of ternary blends for targeted nutritional optimization [39,44].

It is important to acknowledge certain limitations in the present experimental design. The absence of a 100% wheat flour baked cookie control means that the comparative sensory and textural superiority of the alternative formulations is assessed relative to the lowest substitution level (T1) rather than a conventional commercial baseline. Furthermore, because amaranth and chickpea flours were incorporated exclusively in a fixed 1:1 ratio across all treatments, the specific individual techno-functional contributions or potential isolated physical interactions of each alternative flour cannot be entirely decoupled. Future studies incorporating single-flour substitution models alongside a 100% wheat control are necessary to isolate these specific mechanistic variables.

## 5. Conclusions

This study successfully demonstrates that the massive substitution of wheat flour with a binary blend of amaranth and chickpea flours is a highly effective strategy for the nutritional enrichment of short-dough cookies. Thermo-mechanical profiling via Mixolab revealed that a 70% substitution level (T3) profoundly disrupts the dough’s microstructural dynamics. The thermodynamic dilution of the glutenin–gliadin network and the competitive hydration exerted by legume globulins and pseudocereal fibers led to a significant reduction in dough stability (5.40 min) and restricted starch gelatinization (C3 = 0.916 Nm).

However, the multivariate approach employed in this research proves that these rheological constraints do not compromise the end-product in non-fermented matrices. On the contrary, the T3 formulation achieved a superior nutritional density, maximizing crude protein (14.61%) and mineral content (2.18%). Crucially, the integration of Texture Profile Analysis (TPA) and sensory evaluation demonstrated that the significant increase in instrumental hardness (68.09 N) did not negatively affect consumer perception but rather resulted in a highly acceptable texture profile. Consequently, the T3 formulation received the highest overall sensory acceptability (4.07/5.0).

Ultimately, these findings provide useful insights for the food industry in the rational design of plant-based snacks, while acknowledging the need for further validation across broader formulation ranges and commercial-scale conditions. By utilizing regional crops such as Andean amaranth and chickpea, our findings suggest that dough rheological instability does not necessarily preclude the development of products with acceptable sensory outcomes. This research validates that Mixolab parameters can effectively guide the rational design of plant-based, gluten-reduced snacks that meet both modern nutritional demands and stringent consumer organoleptic expectations.

## Figures and Tables

**Table 1:** Physicochemical results of raw materials (chickpea and amaranth flours).

Parameters (%)	Wheat Flour (WF) *	Chickpea Flour (CF)	Amaranth Flour (AF)
Protein	10.50	16.79 ± 0.15 B	18.18 ± 0.12 A
Ash	0.65	3.73 ± 0.10 B	4.85 ± 0.08 A
Moisture	12.10	8.46 ± 0.08 A	9.03 ± 0.04 B
Fat	1.20	4.53 ± 0.12 B	6.23 ± 0.15 A
Acidity	0.08	0.02 ± 0.01 B	0.06 ± 0.01 A

Note: Values for CF and AF are means ± standard deviations calculated from three independent production batches. Different letters in the same row indicate significant differences (*p* < 0.05). * WF data are theoretical reference values for standard commercial refined wheat flour compliant with NTE INEN 616 [30], included solely for baseline comparative purposes.

**Table 2:** Rheological parameters and Profiler indices of the ternary doughs.

Treatment	Water Abs. (%)	C1 Torque (Nm)	Stability (min)	C3 Torque (Nm)	C5 Torque (Nm)	Profiler Index
T1 (30% Sub)	56.3 ± 0.45 A	1.144 ± 0.02 A	9.80 ± 0.15 A	1.291 ± 0.05 A	1.478 ± 0.08 A	2-51-253
T2 (50% Sub)	55.8 ± 0.38 A	1.130 ± 0.01 A	5.00 ± 0.20 B	1.040 ± 0.04 B	1.379 ± 0.06 B	2-41-153
T3 (70% Sub)	49.7 ± 0.52 B	1.108 ± 0.01 B	5.40 ± 0.12 B	0.916 ± 0.03 C	1.396 ± 0.05 B	0-31-154

Note: T1: 15% AF + 15% CF; T2: 25% AF + 25% CF; T3: 35% AF + 35% CF. Values represent the mean of triplicate analyses. Different letters in the same column indicate significant differences (*p* < 0.05).

**Table 3:** Proximate composition and physicochemical properties of the cookies.

Parameter	T1 (30% Sub)	T2 (50% Sub)	T3 (70% Sub)
Protein (%)	13.47 ± 0.15 C	13.67 ± 0.12 B	14.61 ± 0.18 A
Ash (%)	1.35 ± 0.07 C	1.65 ± 0.10 B	2.18 ± 0.05 A
Moisture (%)	7.16 ± 0.13 C	7.39 ± 0.08 B	8.18 ± 0.15 A
Fat (%)	10.88 ± 0.41 C	12.13 ± 0.18 B	13.10 ± 0.19 A
pH	6.72 ± 0.02 C	6.77 ± 0.02 B	6.82 ± 0.03 A

Note: T1: 15% AF + 15% CF; T2: 25% AF + 25% CF; T3: 35% AF + 35% CF. Values are means ± standard deviations calculated from three independent production batches. Different letters in the same row indicate significant differences (*p* < 0.05).

**Table 4:** Instrumental Texture Profile Analysis (TPA) of the formulated cookies.

Treatment	Hardness C1 (N)	Fracturability (N)	Hardness C2 (N)	Chewiness (mJ)
T1 (30% Sub)	32.31 ± 1.28 A	32.31 ± 1.28 A	11.24 ± 2.73 A	3.67 ± 1.20 A
T2 (50% Sub)	53.05 ± 2.28 B	53.05 ± 2.28 B	25.88 ± 1.71 B	17.77 ± 1.01 B
T3 (70% Sub)	68.09 ± 1.54 C	68.09 ± 1.54 C	33.87 ± 1.19 C	25.47 ± 4.48 C

Note: T1: 15% AF + 15% CF; T2: 25% AF + 25% CF; T3: 35% AF + 35% CF. Values are means ± standard deviations. Different letters in the same column indicate significant differences according to ANOVA and Tukey’s test (*p* < 0.05).

**Table 5:** Sensory attributes and consumer acceptance of the enriched cookies.

Attribute	T1 (30% Sub)	T2 (50% Sub)	T3 (70% Sub)
Color	3.0 C	4.0 B	4.0 A
Odor	3.0 B	4.0 B	4.0 A
Flavor	4.0 AB	4.0 B	4.0 A
Texture	3.0 AB	3.0 B	4.0 A
Overall Acceptability	4.0 A	4.0 A	4.0 A

Note: T1: 15% AF + 15% CF; T2: 25% AF + 25% CF; T3: 35% AF + 35% CF. Values are expressed as medians based on a 5-point hedonic scale. Different uppercase letters in the same row indicate significant differences based on the non-parametric Friedman test and Wilcoxon signed-rank post hoc test with Bonferroni correction (*p* < 0.05).

## Data Availability

The original contributions presented in this study are included in the article/Supplementary Materials. Further inquiries can be directed to the corresponding author.

## Supplementary Materials

The following supporting information can be downloaded at https://www.mdpi.com/article/10.3390/foods15152751/s1, Figure S1: Pearson correlation heatmap of physicochemical, textural, and sensory attributes; Table S1: Pearson correlation matrix of physicochemical, textural, and sensory attributes.

## Abbreviations

The following abbreviations are used in this manuscript:

AF	Amaranth Flour
ANOVA	Analysis of Variance
AOAC	Association of Official Analytical Chemists
CF	Chickpea Flour
C1–C5	Mixolab Torque Parameters
HSD	Honestly Significant Difference
NTE INEN	Norma Técnica Ecuatoriana (Ecuadorian Technical Standard)
PCA	Principal Component Analysis
TPA	Texture Profile Analysis
WF	Wheat Flour
