# Supplementary material for: Physicochemical, Rheological, and Sensory Characterization of Gluten-Reduced Cookies Enriched with Amaranth and Chickpea Flours
Source: Foods. 2026 Aug 5;15(15):2751. doi: 10.3390/foods15152751 (PMC13464605; doi:10.3390/foods15152751)

foods

MDPI

Supplementary Material

Table S1. Pearson correlation matrix of physicochemical, textural, and sensory attributes.

Variable	Protein (%)	Ash (%)	Hardness C1 (N)	Acceptability	Texture
Protein (%)	1.00	0.98	0.90	1.00	0.78
Ash (%)	0.98	1.00	0.97	0.99	0.64
Hardness C1 (N)	0.90	0.97	1.00	0.93	0.43
Acceptability	1.00	0.99	0.93	1.00	0.73
Texture	0.78	0.64	0.43	0.73	1.00

Note: Values represent Pearson correlation coefficients (r). Significance was determined at $p < 0.05$.

Figure S1. Pearson correlation heatmap of physicochemical, textural, and sensory attributes.

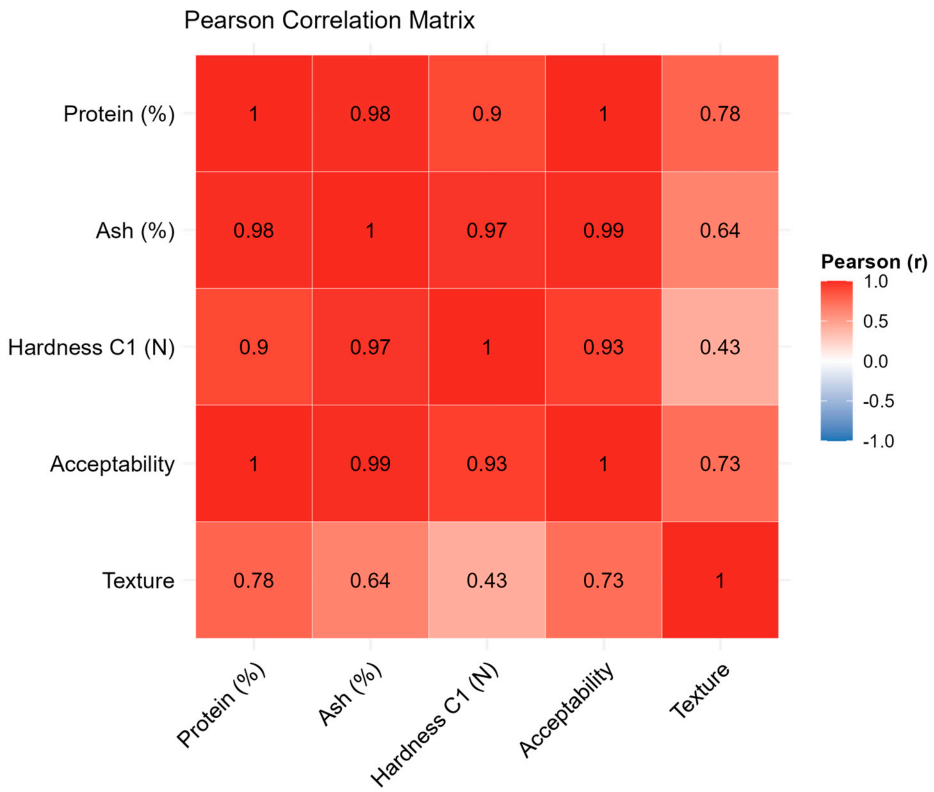

Supplement: Supplementary file 1 [file foods-15-02751-s001.zip › foods-4427541-supplementary.pdf]
